# HIV/AIDS Awareness among VCT Clients: A Cross-Sectional Study from Delhi, India

**DOI:** 10.1155/2014/269404

**Published:** 2014-06-04

**Authors:** Bhanu Mehra, Sonali Bhattar, Preena Bhalla, Deepti Rawat

**Affiliations:** Department of Microbiology, Maulana Azad Medical College, New Delhi 110002, India

## Abstract

The contribution of India to the global burden of HIV/AIDS is significant. A major barrier that the country has faced in its battle against this disease is the inadequate and inaccurate information about it among the population. The present analysis explores the knowledge about HIV/AIDS among clients attending a voluntary counselling and testing (VCT) facility in India. Two hundred clients attending the VCT facility were assessed in this regard using a structured predesigned questionnaire. Sixty-three (31.5%) of the respondents had never heard of HIV/AIDS. In comparison to males, a significantly higher number of females had not heard about the disease (*P* < 0.01). Lower levels of education of participants were found to be significantly associated with the response of not having heard of HIV/AIDS (*P* < 0.01) as was an occupation status of being an unemployed man/housewife (*P* = 0.002). For the 137 (68.5%) respondents who had heard about HIV/AIDS, television was the source of information in 130 (94.9%) followed by posters in 93 (67.9%) and newspapers in 88 (64.2%). While the knowledge about HIV transmission and prevention was good, the extent of misconceptions was high (61.8%). Our study highlights the strong need to raise the levels of HIV awareness among Indian population.

## 1. Introduction

Human immunodeficiency virus (HIV)/acquired immunodeficiency syndrome (AIDS) is a major and one of the most serious public health challenges in today's world. An estimated 35.3 million people across the world are infected with HIV [1]. According to the joint United Nations programme on HIV/AIDS (UNAIDS), HIV is one of the leading causes of mortality across the globe [2]. A major proportion of HIV infected individuals reside in the developing world, of which the populous countries of the Asian subcontinent are of major concern.

The contribution of India to the global burden of HIV/AIDS is significant with nearly 2.39 million people currently affected with the disease in the country [3]. Though the HIV prevalence in India is low, the large population base of the country explains this enormous absolute number of HIV infected. The large population base also makes the country more vulnerable to HIV. In addition several other factors contribute to this vulnerability including low levels of education, poverty, early age of sexual debut, limited access to health services especially in rural settings, inadequate information about modes of HIV transmission, and misconceptions and myths revolving around HIV/AIDS [4].

Among these a major barrier in the battle against HIV/AIDS has been inadequate and inaccurate information that further perpetuates various forms of social stigmas and discriminations against the HIV infected. The synonymous interpretation of HIV/AIDS to immorality would result in hesitation on part of the people to get themselves tested for HIV infection leaving a large number of HIV infected individuals unaware of their status. This could be potentially devastating as it would accelerate the unknowing, unchecked, and silent transmission of HIV infection from the infected to the naive population. Low levels of education would also leave the population unaware of the HIV risk reduction strategies. Another matter of concern is the changing face of HIV epidemic in India. The epidemic has crossed the conventional boundaries of the traditional high risk groups where it initially started and has now percolated into the general population [5]. Undoubtedly, India has emerged as the new epicentre of this international epidemic.

A bipronged approach has been employed to limit the disastrous consequences of this epidemic. The first strategy is promoting HIV awareness and knowledge among the population through media as well as through voluntary counselling and testing (VCT) facilities so as to empower the individuals to protect themselves by adopting safe sexual practices and other necessary precautionary measures. The second approach is increased and easy accessibility to antiretroviral therapy to reduce the mortality due to HIV/AIDS. Though antiretroviral drugs can slow down the progression of this disease, they are not curative. Moreover, an effective HIV vaccine is also far from reality. Therefore the only feasible and cost-effective approach in the current situation, especially in the developing world, would be to disseminate among the general population correct, accurate, and complete knowledge about HIV/AIDS transmission modes, risk factors, preventive measures, and available therapeutic options. In this context, education has often been described as a “social vaccine” with information, education, and communication (IEC) being considered the key tools of HIV prevention.

A number of studies have attempted to explore the peoples' perceptions about HIV/AIDS. The present analysis was undertaken as an attempt to assess the extent of knowledge about HIV/AIDS among clients attending a VCT facility in India and to correlate their levels of awareness with various sociodemographic determinants.

## 2. Materials and Methods

The present cross-sectional analysis was conducted at the VCT facility of a tertiary care health centre situated in Delhi, India. The VCT caters to nearly 1100 clients per month. The study population is comprised of clients attending the VCT facility in November-December 2013. Two hundred consecutive clients who were 18 years of age and above and who verbally consented to participate in the interviews after the primary purpose of the study was explained to them were enrolled in the analysis. The interviews were conducted by the investigators before the clients underwent the HIV counselling sessions. All the interviews were voluntary and confidential.

A standardised, structured predesigned questionnaire was employed and was translated into Hindi, which is the commonly used and fully understood language of this region. The same procedure was followed by the interviewers for all the participants whether literate or nonliterate and the responses were recorded. The interviews were conducted independently by two senior resident doctors and the quality of the questionnaire and of the personal interviews was assessed by the faculty in charge of the VCT. The questionnaire is comprised of two broad sections. The first section dealt with the sociodemographic characteristics of the participants (age, sex, education, marital status, type of family, occupation, and place of residence). While “nuclear family” was defined as a household consisting of a father, a mother, and their children all in one household dwelling, the term “joint family” was used to describe a family composed of parents, their children, and the children's spouses and offspring all in one household. The names of the participants were not entered so as to maintain anonymity and confidentiality.

The second section of the questionnaire sought to explore the participants' awareness regarding various aspects of HIV/AIDS. This section comprised only close-ended questions, the answers to which were recorded as yes/no/I do not know. The participants were initially questioned if they had ever heard of HIV/AIDS. Only those respondents who claimed to have heard of HIV/AIDS were questioned further. These participants were asked about the source of their information about HIV/AIDS. Their level of awareness regarding modes of HIV transmission was judged by their ability to correctly identify the four principal ways by which this infection can spread. By incorporating misleading questions in the panel, we attempted to simultaneously explore and identify some common misconceptions that prevail among Indian population pertaining to modes of HIV transmission. In the subsequent questions the participants' perception regarding availability of possible treatment options and their knowledge about preventive measures/practices of HIV/AIDS were assessed.

For data analysis of responses to the questions concerning modes of transmission, treatment, and prevention of HIV/AIDS, participants with an “I do not know” response were also considered unaware about the concerned aspect as were the participants with a “No” response. For analyzing the responses to the panel of questions dealing with misconceptions, respondents with “No” response to all the questions were categorized as having “no misconception” while those with a “Yes” response to even one of the questions were categorized as having “at least one misconception.”

Data entry was performed using Microsoft Excel sheet. All the entries were doubly checked for any possible keyboard errors. Data was analyzed using the Epi Info software, and Chi-square and Fischer's exact test were applied to determine the difference of proportion between qualitative variables. A *P* value less than 0.05 was considered as statistically significant.

## 3. Results

Of the 200 study subjects included in this analysis, 46 (23%) were 18–24 years, 75 (37.5%) were 25–31 years, 43 (21.5%) were 32–38 years, 25 (12.5%) were 39–45 years, 3 (1.5%) were 46–50 years, and 8 (4%) were more than 50 years of age. The mean age of the study participants was 30.85 ± 9.10 years with a range of 18–62 years. Eighty (40%) of the 200 participants were males and 120 (60%) were females. One hundred and sixty (80%) of the respondents were married while 40 (20%) were unmarried. Fifty-eight (29%) of the participants were nonliterate, 68 (34%) were educated up to primary school level, 50 (25%) were educated up to secondary school level, and only 24 (12%) had an education level of college and above. Majority of the respondents, that is, 117 (58.5%), were unemployed men or housewives, 25 (12.5%) were daily wagers, 39 (19.5%) were salaried, and 19 (9.5%) belonged to business class. Of the 200 respondents who participated, 88 (44%) belonged to rural areas while 112 (56%) resided in urban localities. Majority of the respondents (54%) were from nuclear families and the remaining (46%) belonged to joint families. The sociodemographic profile of the study population is summarised in Table 1.

Sixty-three (31.5%) of the respondents admitted that they had never heard of HIV/AIDS. The percentage of females (42.5%) who had never heard of HIV/AIDS was significantly higher than the corresponding percentage of males (15%) (*P* < 0.01). A statistically significant association was also noted between the response “not heard about HIV/AIDS” and the education level of the participants (*P* < 0.01) and we observed that the percentage of respondents who had not heard about HIV/AIDS declined steadily from 63.8% among nonliterates to 30.9% among primary school educated respondents, 10% among secondary school educated respondents, and 0% among those with educational qualification of college and above. With regard to occupation, we observed that a significantly higher proportion of unemployed respondents/housewives (40.2%) had not heard of HIV/AIDS (*P* = 0.002) while a significantly higher proportion of participants belonging to salaried class (87.2%) had heard about HIV/AIDS (*P* = 0.009) (Table 2).

Among the 137 (68.5%) respondents who had heard about HIV/AIDS, television was the main source of information for 130 (94.9%), posters for 93 (67.9%), and newspapers for 88 (64.2%). A conversation or discussion with friends, health professionals, and teachers was a possible source of information about HIV/AIDS in 45 (32.8%), 34 (24.8%), and 26 (19%) respondents, respectively. A conversation or discussion within family was the least reported source of information (8%) (Table 3).

Of the 137 study participants who were further interviewed, 135 (98.5%) could identify unprotected sex as the mode of HIV transmission. One hundred thirty-two (96.4%) were aware of blood transfusion and use of unsterile needles and syringes as other modes of HIV transmission. Only 122 (89.1%) knew about HIV transmission from infected mother to her child (Table 4). On correlating various sociodemographic variables with knowledge about each of the above mentioned principal modes of HIV transmission, we observed that awareness about unsafe sex as a possible mode was significantly higher among married participants (*P* = 0.049). A statistically significant association was also noted between knowledge of unsterile needles and syringes as a transmission mode and education level (nonliterate versus primary versus secondary versus college and above; *P* = 0.024) and urban residence (*P* = 0.01). Likewise a statistically significant difference was noted between the knowledge of married respondents about mother to baby transmission of HIV infection and their unmarried counterparts (*P* = 0.043) (Table 5).

On exploring the extent of misconceptions about HIV transmission, we observed that 45 (32.9%) had a false perception that HIV can be transmitted by coughing/sneezing; 47 (34.3%) thought that it can be transmitted by mosquito bite; 20 (14.6%) thought that it can be transmitted by touching an HIV infected person; 26 (19%) thought that it can be transmitted by working with an HIV infected person, while 32 (23.3%) and 41 (29.9%) incorrectly stated that it can be transmitted by eating with and by sharing towels/clothes/handkerchief of a person with HIV/AIDS, respectively (Table 4). On redefining the criteria of misconceptions as having “at least one misconception” we observed that 84 (61.8%) of 136 respondents had misconceptions regarding HIV transmission (one respondent with “do not know” response to all the questions in this panel was excluded from the analysis). On further correlation with sociodemographic determinants, we noted that a statistically significant association existed between the presence of misconceptions and the education status of the respondents (*P* < 0.01) with a declining trend observed in the presence of misconceptions as the education level of the respondents increased (81% in nonliterate participants, 78.3% in primary school educated participants, 46.7% in secondary school educated participants, and 41.7% in those educated till college and above).

Of the 137 participants who had heard about HIV/AIDS, only 68 (49.6%) were aware of the availability of antiretroviral treatment (Table 4). Females seemed to be more aware regarding availability of antiretroviral treatment than males (63.8% versus 35.3%; *P* = 0.002). Likewise married respondents seemed to more aware about this aspect than their unmarried counterparts (54.7% versus 32.3%; *P* = 0.045).

Use of condom as a preventive intervention was known to 136 (99.3%) respondents. All (100%) participants understood the role of loyalty to a single sexual partner and use of sterile disposable needles and syringes as precautionary measures to protect themselves from contracting HIV/AIDS. One hundred thirty-six (99.3%) knew the importance of screening of blood before transfusion. No statistically significant correlation could be established between sociodemographic profile of the study participants and their awareness regarding HIV prevention strategies.

## 4. Discussion

In the present study, 68.5% of the participants had heard about HIV/AIDS. Similar findings were observed in a community based cross-sectional study conducted among rural youth of Saurashtra where nearly two-thirds of the respondents had ever heard about this infection [6]. Mass media (television, posters, and newspapers) followed by friends or peer group were the main sources of information for the respondents in our study. Our findings are in broad agreement with those of a study conducted by Subramaniam et al. that has reported mass media and friends as the chief sources of information on HIV/AIDS among rural south Indian women of Tamil Nadu [7]. Similar findings were reported in another study conducted in the underprivileged population of Chandigarh [8]. Television was also reported as the most important source of information about HIV/AIDS among medical and allied health science students of Bhubaneswar, Odisha [9]. Numerous other studies also highlight the pivotal role that mass media have played in creating HIV awareness among Indian population [10, 11]. As with another study conducted in Raigad district, we also observed that discussion in family is a less common source of information about HIV and other sexual issues in Indian population [12]. Our findings show that the conservative atmosphere of the Indian society with its social restrictions and customs prevents free and open discussion about HIV/AIDS within family.

While majority of the respondents who had heard about HIV/AIDS had a good knowledge about HIV transmission modes, nearly 11% were still unaware of risk of HIV transmission from infected mother to her baby. Our findings are consistent with another study conducted among rural youth of Saurashtra, Gujarat, where the participants were less aware of mother to child transmission of HIV in comparison to other modes [6]. This lacuna in knowledge about HIV/AIDS among the Indian population needs to be addressed and stressed upon so as to encourage more and more women to undergo antenatal HIV testing and to ensure timely initiation of antiretroviral therapy in the pregnant women who are identified as HIV positive.

As with other studies conducted in Indian population, we observed that compared to males, a lower percentage of females have heard about HIV/AIDS. This finding highlights that members of the biologically more susceptible gender are largely unaware of the disease to which they are highly vulnerable, a phenomenon driven by the male dominated Indian society that denies the females education and access to information [13].

Education was found to have a direct relation to the awareness levels of the respondents. Not only were the nonliterate subjects less likely to have heard about HIV/AIDS, but also the awareness of some aspects of HIV transmission such as spread by sharing of needles and syringes was significantly lower in the nonliterate subjects. A study conducted in rural district of Raigad also found the level of awareness to be significantly lower in illiterates as compared to literates [12]. Malleshappa et al. have also reported literates to have better knowledge of HIV/AIDS than nonliterates [14]. Education not only increases the accessibility to information but also enables better understanding and interpretation of the informative material and eventually its reflection in one's personal risk reduction strategies.

With regard to occupation, we observed that a significantly lower number of unemployed men and housewives had heard of HIV/AIDS while the proportion of respondents from the salaried class who had heard about it was significantly higher. Differences in knowledge between different occupational groups have also been highlighted in another study conducted in Hyderabad where students and people in service and business were found to have high awareness levels while lowest awareness levels were seen in housewives, cultivators, agriculture labourers, and industrial workers [15].

While we could not establish a correlation between age of respondents and HIV awareness, lower awareness levels among younger populations have been reported in other studies [12].

Married respondents seemed to be more aware of certain aspects of HIV transmission such as unsafe sex and mother to child HIV transmission as well as antiretroviral treatment. Higher awareness levels in married as compared to unmarried respondents have also been documented in other studies [12].

While the knowledge of HIV transmission modes and preventive strategies was good among the respondents who were interviewed, the prevalence of misconceptions regarding HIV transmission was high. Our findings are consistent with other studies where casual contact, mosquito bite, bed bugs, public toilets, pools, and sharing meals have been considered as modes of HIV transmission by the participants [6, 14, 16–18]. As with other studies misconceptions were more among the respondents who were nonliterate [14, 19, 20]. A summary of some of the previous similar studies undertaken in different demographic regions of the Indian subcontinent is given in Table 6.

## 5. Conclusion

Our study highlights that a significant proportion of Indian population is unaware of HIV/AIDS to the extent that they have not even heard of it. There is a strong need to raise the levels of HIV awareness among the population especially among women and nonliterate members of the community. The prevalence of misconceptions regarding HIV transmission is also high in the Indian population. While media has played a crucial role in attaining the present level of knowledge about HIV/AIDS in the community, much efforts are still needed in this direction including education in conjunction with evolution of novel creative strategies to reach out to more and more people, make them aware about HIV/AIDS, improve their existing knowledge about this disease, and demystify their myths and misconceptions.

## Figures and Tables

**Table 1:** Sociodemographic profile of study population.

Sociodemographic variable	Number (*n* = 200)	Percentage (%)
Age group (years)		
18–24 years	46	23%
25–31 years	75	37.5%
32–38 years	43	21.5%
39–45 years	25	12.5%
46–50 years	3	1.5%
>50 years	8	4%
Gender		
Male	80	40%
Female	120	60%
Marital status		
Single	40	20%
Married	160	80%
Education		
Nonliterate	58	29%
Primary	68	34%
Secondary	50	25%
College and above	24	12%
Occupation		
Unemployed/housewives	117	58.5%
Daily wages	25	12.5%
Salaried	39	19.5%
Business	19	9.5%
Type of residence		
Rural	88	44%
Urban	112	56%
Type of family		
Nuclear	108	54%
Joint	92	46%

**Table 2:** Distribution of participants depending on whether they have “heard of HIV/AIDS” or “not” (*n* = 200).

Sociodemographic determinants	Not heard about HIV/AIDS (*n* = 63)	Heard about HIV/AIDS (*n* = 137)	*P* value
Age group (years)			
18–24 years	15 (32.6%)	31 (67.4%)	0.027; S
25–31 years	17 (22.7%)	58 (77.3%)	
32–38 years	13 (30.2%)	30 (69.8%)	
39–45 years	13 (52%)	12 (48%)	
46–50 years	0 (0%)	3 (100%)	
>50 years	5 (62.5%)	3 (37.5%)	
Gender			
Male	12 (15%)	68 (85%)	<0.01; S
Female	51 (42.5%)	69 (57.5%)	
Marital status			
Single	9 (22.5%)	31 (77.5%)	0.238; NS
Married	54 (33.8%)	106 (66.2%)	
Education			
Nonliterate	37 (63.8%)	21 (36.2%)	<0.01; S
Primary	21 (30.9%)	47 (69.1%)	
Secondary	5 (10%)	45 (90%)	
College and above	0 (0%)	24 (100%)	
Occupation			
Unemployed/ housewives	47 (40.2%)	70 (59.8%)	0.002; S
Daily wages	8 (32%)	17 (68%)	0.862; NS
Salaried	5 (12.8%)	34 (87.2%)	0.009; S
Business	3 (15.8%)	16 (84.2%)	0.197; NS
Type of residence			
Rural	32 (36.4%)	56 (63.6%)	0.246; NS
Urban	31 (27.7%)	81 (72.3%)	
Type of family			
Nuclear	37 (34.3%)	71 (65.7%)	0.448; NS
Joint	26 (28.3%)	66 (71.7%)	

HIV: human immunodeficiency virus; AIDS: acquired immunodeficiency syndrome; S: significant; NS: nonsignificant.

**Table 3:** Sources of information regarding HIV/AIDS (*n* = 137).

Source of information	Number	Percentage
Television	130	94.9%
Newspapers	88	64.2%
Family	11	8%
Friends	45	32.8%
Teachers	26	19%
Doctors	34	24.8%
Posters	93	67.9%

HIV: human immunodeficiency virus; AIDS: acquired immunodeficiency syndrome.

**Table 4:** Knowledge of participants regarding various aspects of HIV/AIDS.

Question	Response		
	Yes	No	Do not know
Have you ever heard about HIV/AIDS? (*n* = 200)	137 (68.5%)	63 (31.5%)	—

Knowledge regarding modes of HIV transmission: (*n* = 137)			
(a) Unprotected sexual intercourse with HIV infected	135 (98.5%)	2 (1.5%)	—
(b) Transfusion of infected blood	132 (96.4%)	1 (0.7%)	4 (2.9%)
(c) Use of unsterile needles/syringes	132 (96.4%)	2 (1.4%)	3 (2.2%)
(d) From an HIV infected mother to her baby	122 (89.1%)	4 (2.9%)	11 (8%)

Myths and misconceptions regarding HIV transmission: (*n* = 137)			
Do you think HIV/AIDS can be transmitted by			
(a) Coughing/sneezing?	45 (32.9%)	75 (54.7%)	17 (12.4%)
(b) Mosquito bite?	47 (34.3%)	73 (53.3%)	17 (12.4%)
(c) Touching a person with HIV/AIDS?	20 (14.6%)	110 (80.3%)	7 (5.1%)
(d) Working with a person with HIV/AIDS?	26 (19%)	107 (78.1%)	4 (2.9%)
(e) Taking food with a person with HIV/AIDS?	32 (23.3%)	99 (72.3%)	6 (4.4%)
(f) Sharing towels/clothes/handkerchief of a person with HIV/AIDS?	41 (29.9%)	82 (59.9%)	14 (10.2%)

Knowledge regarding availability of antiretroviral treatment (*n* = 137)	68 (49.6%)	19 (13.9%)	50 (36.5%)

Knowledge regarding preventive interventions for HIV/AIDS: (*n* = 137)			
(a) Use of condom during sexual intercourse	136 (99.3%)	—	1 (0.7%)
(b) Loyalty to a single partner	137 (100%)	—	—
(c) Use of sterile disposable needles and syringes	137 (100%)	—	—
(d) Transfusion of screened and tested blood units	136 (99.3%)	1 (0.7%)	—

HIV: human immunodeficiency virus; AIDS: acquired immunodeficiency syndrome.

**Table 5:** Distribution of participants according to their knowledge regarding modes of HIVAIDS transmission (*n* = 137).

Sociodemographic variable	Unprotected sexual intercourse with HIV infected (*n* = 137)			Transfusion of infected blood (*n* = 137)			Use of unsterile needles/syringes (*n* = 137)			From an HIV infected mother to her baby (*n* = 137)		
	Aware (*n* = 135)	Unaware (*n* = 2)	*P* value	Aware (*n* = 132)	Unaware (*n* = 5)	*P* value	Aware (*n* = 132)	Unaware (*n* = 5)	*P* value	Aware (*n* = 122)	Unaware (*n* = 15)	*P* value
Age group (years)												
18–24 years	29 (93.5%)	2 (6.5%)	0.225; NS	29 (93.5%)	2 (6.5%)	0.808; NS	30 (96.8%)	1 (3.2%)	0.962; NS	25 (80.6%)	6 (19.4%)	0.301; NS
25–31 years	58 (100%)	0 (0%)		57 (98.3%)	1 (1.7%)		56 (96.6%)	2 (3.4%)		52 (89.7%)	6 (10.3%)	
32–38 years	30 (100%)	0 (0%)		29 (96.7%)	1 (3.3%)		29 (96.7%)	1 (3.3%)		29 (96.7%)	1 (3.3%)	
39–45 years	12 (100%)	0 (0%)		11 (91.7%)	1 (8.3%)		11 (91.7%)	1 (8.3%)		11 (91.7%)	1 (8.3%)	
46–50 years	3 (100%)	0 (0%)		3 (100%)	0 (0%)		3 (100%)	0 (0%)		2 (66.7%)	1 (33.3%)	
>50 years	3 (100%)	0 (0%)		3 (100%)	0 (0%)		3 (100%)	0 (0%)		3 (100%)	0 (0%)	
Gender												
Male	68 (100%)	0 (0%)	0.496; NS	68 (100%)	0 (0%)	0.057; NS	66 (97.1%)	2 (2.9%)	1; NS	63 (92.6%)	5 (7.4%)	0.287; NS
Female	67 (97.2%)	2 (2.8%)		64 (92.8%)	5 (7.2%)		66 (95.7%)	3 (4.3%)		59 (85.5%)	10 (14.5%)	
Marital status												
Single	29 (93.6%)	2 (6.4%)	0.049; S	30 (96.8%)	1 (3.2%)	1; NS	29 (93.6%)	2 (6.4%)	0.316; NS	24 (77.4%)	7 (22.6%)	0.043; S
Married	106 (100%)	0 (0%)		102 (96.2%)	4 (3.8%)		103 (97.2%)	3 (2.8%)		98 (92.5%)	8 (7.5%)	
Education												
Nonliterate	20 (95.3%)	1 (4.7%)	0.419; NS	19 (90.5%)	2 (9.5%)	0.339; NS	18 (85.7%)	3 (14.3%)	0.024; S	16 (76.2%)	5 (23.8%)	0.081; NS
Primary	47 (100%)	0 (0%)		46 (97.9%)	1 (2.1%)		45 (95.7%)	2 (4.3%)		45 (95.7%)	2 (4.3%)	
Secondary	44 (97.7%)	1 (2.3%)		43 (95.5%)	2 (4.5%)		45 (100%)	0 (0%)		41 (91.1%)	4 (8.9%)	
College and above	24 (100%)	0 (0%)		24 (100%)	0 (0%)		24 (100%)	0 (0%)		20 (83.3%)	4 (16.7%)	
Occupation												
Unemployed/housewives	69 (98.6%)	1 (1.4%)	1; NS	66 (94.3%)	4 (5.7%)	0.366; NS	68 (97.1)	2 (2.9)	0.676; NS	60 (85.7%)	10 (14.3%)	0.314; NS
Daily wages	16 (94.2%)	1 (5.8%)	0.233; NS	16 (94.1%)	1 (5.9%)	0.489; NS	15 (88.2%)	2 (11.8%)	0.116; NS	15 (88.2%)	2 (11.8%)	1; NS
Salaried	34 (100%)	0 (0%)	1; NS	34 (100%)	0 (0%)	0.332; NS	33 (97.1%)	1(2.9%)	1; NS	32 (94.1%)	2 (5.9%)	0.356; NS
Business	16 (100%)	0 (0%)	1; NS	16 (100%)	0 (0%)	1; NS	16 (100%)	0 (0%)	1; NS	15 (93.8%)	1 (6.2%)	1; NS
Type of residence												
Rural	54 (96.5%)	2 (3.5%)	0.165; NS	53 (94.6%)	3 (5.4%)	0.398; NS	51 (91.1%)	5 (8.9%)	0.010; S	47 (83.9%)	9 (16.1%)	0.187; NS
Urban	81 (100%)	0 (0%)		79 (97.5%)	2 (2.5%)		81 (100%)	0 (0%)		75 (92.6%)	6 (7.4%)	
Type of family												
Nuclear	70 (98.6%)	1 (1.4%)	1; NS	67 (94.4%)	4 (5.6%)	0.367; NS	67 (94.4%)	4 (5.6%)	0.367; NS	62 (87.3%)	9 (12.7%)	0.690; NS
Joint	65 (98.5%)	1 (1.5%)		65 (98.5%)	1 (1.5%)		65 (98.5%)	1 (1.5%)		60 (90.9%)	6 (9.1%)	

HIV: human immunodeficiency virus; AIDS: acquired immunodeficiency syndrome; S: significant; NS: nonsignificant.

**Table 6:** A summary of various studies undertaken to explore HIV/AIDS awareness among Indian population.

Authors	Demographic region	Sample size	Sex distribution	Education level of respondents	Occupation/economic status of respondents	Extent of misconceptions about HIV transmission
Srivastava et al. [21]	Bareilly district, Uttar Pradesh	341	Males: 68% Females: 32%	Studying in secondary school at the time of enrolling to study	Students	Mosquito bite (20.5%) Through sweat (19.6%) Eating together (18.2%) Kissing (17.3%) Living together (15.2%) Touching an infected person (13.8%) Sharing same clothes (13.8%) Through common toilet (11.4%) Through working together (9.7%)

Malleshappa et al. [14]	Kuppam Mandal, Andhra Pradesh	850	Males: 53.6% Females: 46.3%	Illiterate: 12.3% Secondary school/below: 34.9% Higher secondary: 40.7% Graduates: 8% Postgraduates: 4%	Student: 38% Agricultural labourer: 43.1% Business: 3% Government Service: 3.6% Housewife: 5.2% Others (including unemployed): 6.8%	Kissing on cheeks (20%) Sharing utensils (20%) Mosquito bite (17%) Public toilets (11.8%)

Yadav et al. [6]	Saurashtra region, Gujarat	1,237	Males: 49.9% Females: 50.1%	Illiterate: 13.42%, Literate: 86.58%	Percentage distribution not mentioned	Living with an HIV infected person: 20.78% Mosquito bite 18.56% Eating food with an HIV infected individual: 17.25%

Shrotri et al. [22]	Pune, Maharashtra	707	All females	Illiterate/primary: 33% Secondary or above: 67%	Housewife: 83% Other: 17%	By mosquito bite: 62% By food: 56% Staying in the same room: 54% Donating blood: 48%

Andersson and Westergren [23]	Solapur district, Maharashtra	260	*Village* Males: 77% Females: 23% *City* Males: 39% Females: 61%	Studying in higher secondary at the time of enrolling to study	Students *Economic situation* Very poor: 3% Poor: 1% Average: 39% Good: 44% Very good: 7% No answer: 6%	HIV spreads by kissing: 36.9% in village versus 31.9% in city HIV spreads by washing or changing clothes for someone who has the infection: 6.6% in village versus 1.4% in city HIV spreads by eating or drinking from the same plates and cups: 3.3% in village versus 2.9% in city Shaking hands/hugging/living in the same house: 2.5% in village versus 0.7% in city

HIV: Human immunodeficiency virus; AIDS: Acquired immunodeficiency syndrome.

## References

[1] UNAIDS Report on the global AIDS epidemic. http://www.unaids.org/en/media/unaids/contentassets/documents/epidemiology/2013/gr2013/UNAIDS_Global_Report_2013_en.pdf.

[2] UNAIDS AIDS epidemic update. http://www.unaids.org/en/KnowledgeCentre/HIVData/EpiUpdate/EpiUpdArchive/2009/default.asp.

[3] Annual Report 2010-11. Department of AIDS Control. National AIDS Control Organization. Ministry of Health & Family Welfare. Government of India. http://www.nacoonline.org/upload/REPORTS/NACO%20Annual%20Report%202010-11.pdf.

[4] Hawkes S, Santhya KG (2002). Diverse realities: sexually transmitted infections and HIV in India. *Sexually Transmitted Infections*.

[5] UNAIDS National response brief-India. www.unaids.org/nationalresponse/result.asp.

[6] Yadav SB, Makwana NR, Vadera BN, Dhaduk KM, Gandha KM (2011). Awareness of HIV/AIDS among rural youth in India: a community based cross-sectional study. *Journal of Infection in Developing Countries*.

[7] Subramanian T, Gupte MD, Ezhil R (2007). AIDS: an understanding in rural women of South-India. *Indian Journal of Sexually Transmitted Diseases*.

[8] Bhatia V, Swami HM, Kaur AP (2004). An intervention study to enhance AIDS awareness among underprivileged population in Chandigarh. *Indian Journal of Dermatology, Venereology and Leprology*.

[9] Chauhan A, Hussain M, Pati S, Nallala S, Mishra J (2012). Knowledge and attitudes related to HIV/AIDS among medical and allied health science students. *Indian Journal of Community Health (IJCH)*.

[10] Tibdewel SS, Wadhva SK (2001). HIV/AIDS awareness among hospital employees. *Indian Journal of Medical Sciences*.

[11] Chatterjee N (1999). AIDS-related information exposure in the mass media and discussion within social networks among married women in Bombay, India. *AIDS Care—Psychological and Socio-Medical Aspects of AIDS/HIV*.

[12] Solat S, Velhal GD, Mahajan H, Rao A, Sharma B (2012). Assessment of awareness about HIV/AIDS and operationalization of interventions in rural population of Raigad district, India. *International Journal of Scientific and Research Publications*.

[13] United Nations Development Programme Gender: impact of HIV and AIDS in India. http://www.undp.org/content/dam/india/docs/gender.pdf.

[14] Malleshappa K, Krishna S, Shashikumar (2012). Awareness and attitude of youth toward HIV/AIDS in rural Southern India. *Biomedical Research*.

[15] Sudha RT, Vijay DT, Lakshmi V (2005). Awareness, attitudes, and beliefs of the general public towards HIV/AIDS in Hyderabad, a capital city from South India. *Indian Journal of Medical Sciences*.

[16] Sikander Q, Malik R, Afzal R (2000). Knowledge, attitude and practices of college students of Rawalpindi regarding HIV/AIDS. *Pakistan Journal of Medical Research*.

[17] Ganguli SK, Rekha PP, Gupte N, Charan UA (2002). AIDS awareness among undergraduate students, Maharashtra. *Indian Journal of Public Health*.

[18] Meundi AD, Amma A, Rao A, Shetty S, Shetty AK (2008). Cross-sectional population-based study of knowledge, attitudes, and practices regarding HIV/AIDS in Dakshina Kannada district of Karnataka, India. *Journal of the International Association of Physicians in AIDS Care*.

[19] Ambati BK, Ambati J, Rao AM (1997). Dynamics of knowledge and attitudes about AIDS among the educated in southern India. *AIDS Care*.

[20] Bhattacharya G, Cleland C, Holland S (2000). Knowledge about HIV/AIDS, the perceived risks of infection and sources of information of Asian-Indian adolescents born in the USA. *AIDS Care—Psychological and Socio-Medical Aspects of AIDS/HIV*.

[21] Srivastava A, Mahmood SE, Mishra P, Shrotriya VP, Shaifali I (2011). Adolescence awareness; a better tool to combat HIV/AIDS. *National Journal of Community Medicine*.

[22] Shrotri A, Shankar AV, Sutar S (2003). Awareness of HIV/AIDS and household environment of pregnant women in Pune, India. *International Journal of STD and AIDS*.

[23] Andersson C, Westergren C (2004). *Still Scant and Insufficient Knowledge about HIV/AIDS among Teenagers in Solapur District, Maharashtra State, India*.

